# Supplementary figures and images for: Proteomic indicators of oxidation and hydration state in colorectal cancer
Source: PeerJ. 2016 Jul 20;4:e2238. doi: 10.7717/peerj.2238 (PMC4958012; doi:10.7717/peerj.2238)

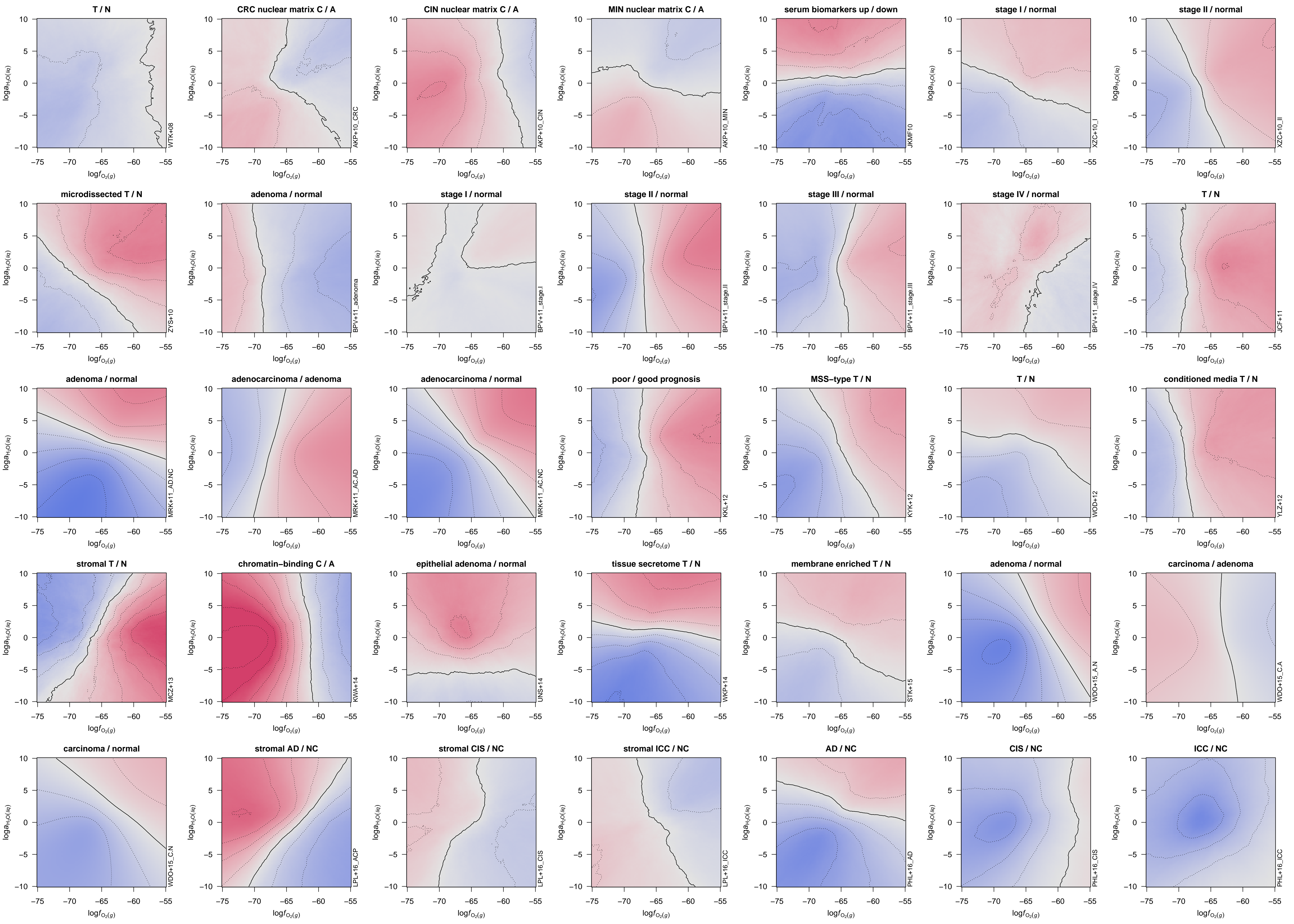

Supplement: Figure S1 [file peerj-04-2238-s002.pdf]

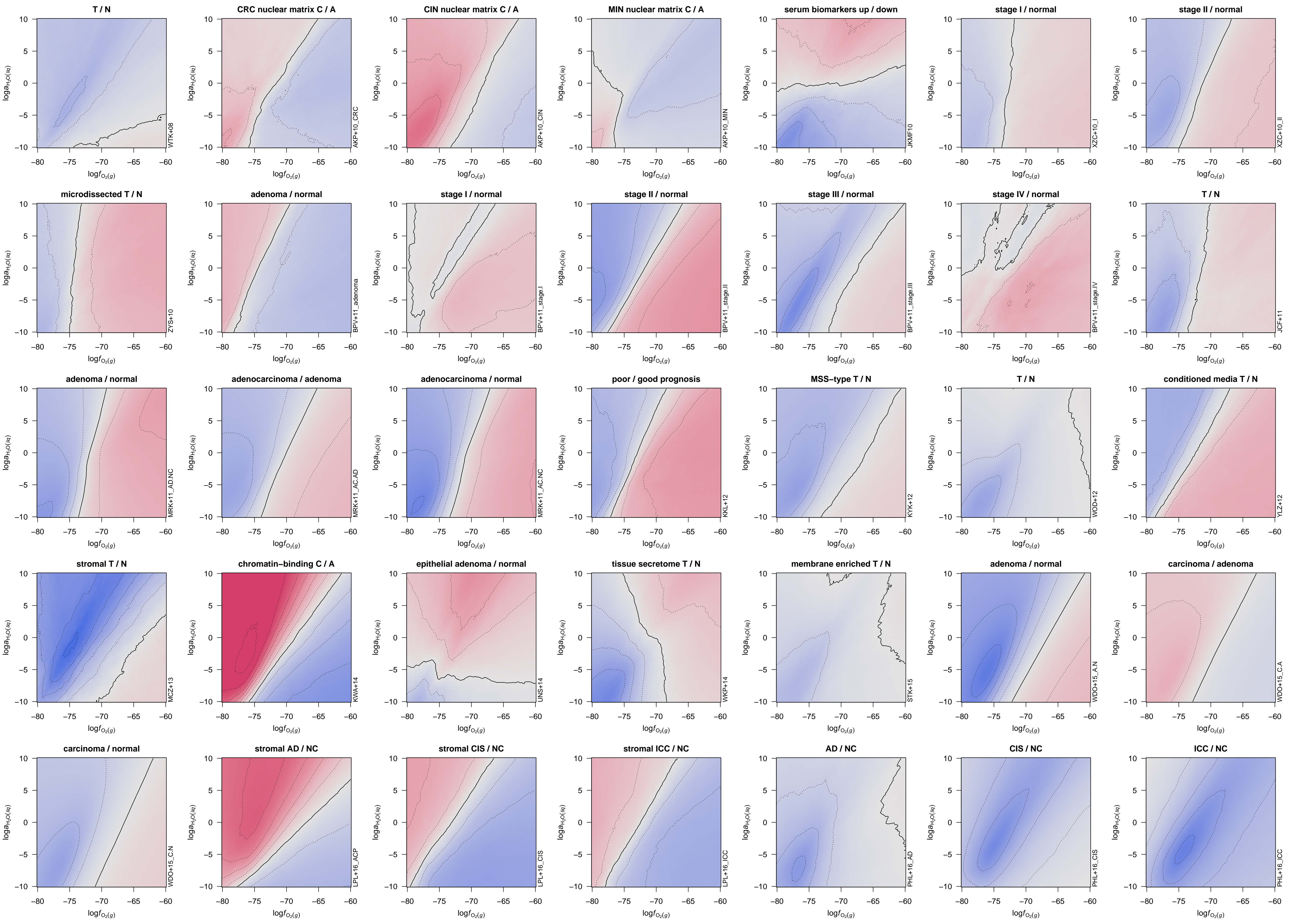

Supplement: Figure S2 [file peerj-04-2238-s003.pdf]

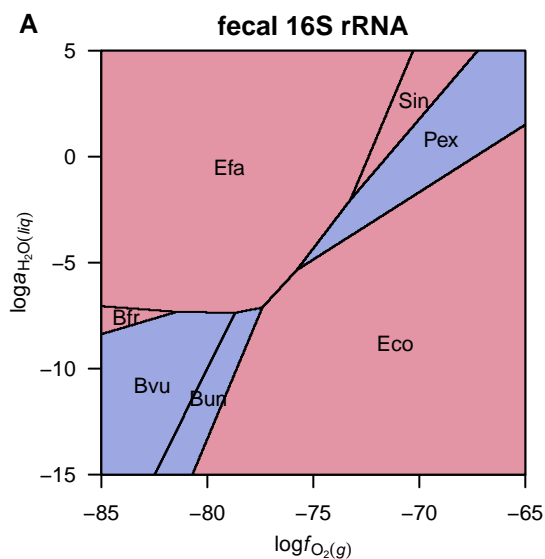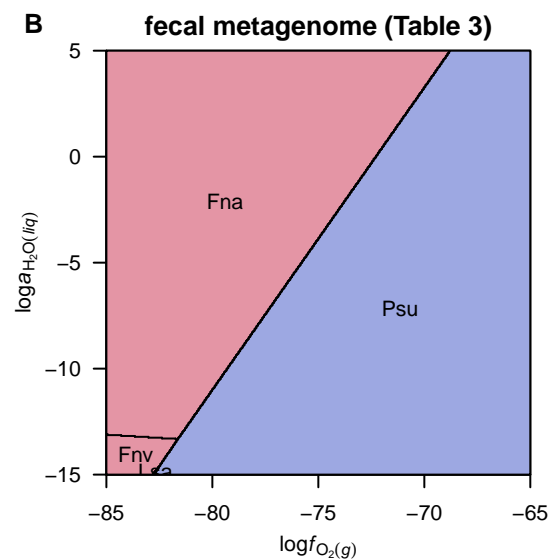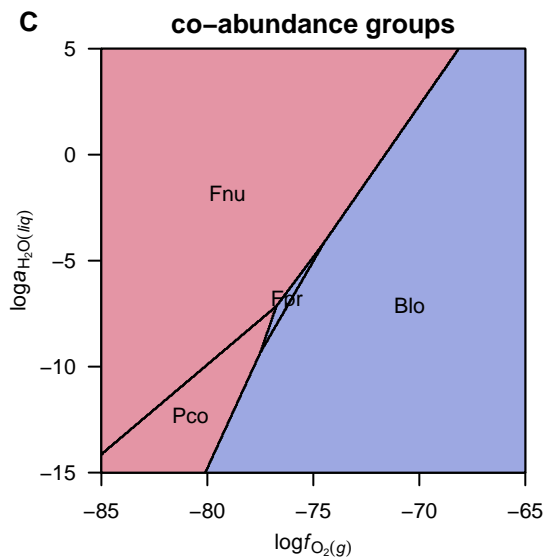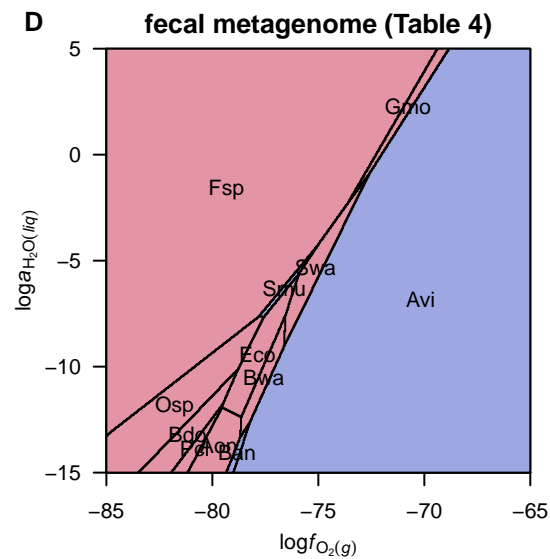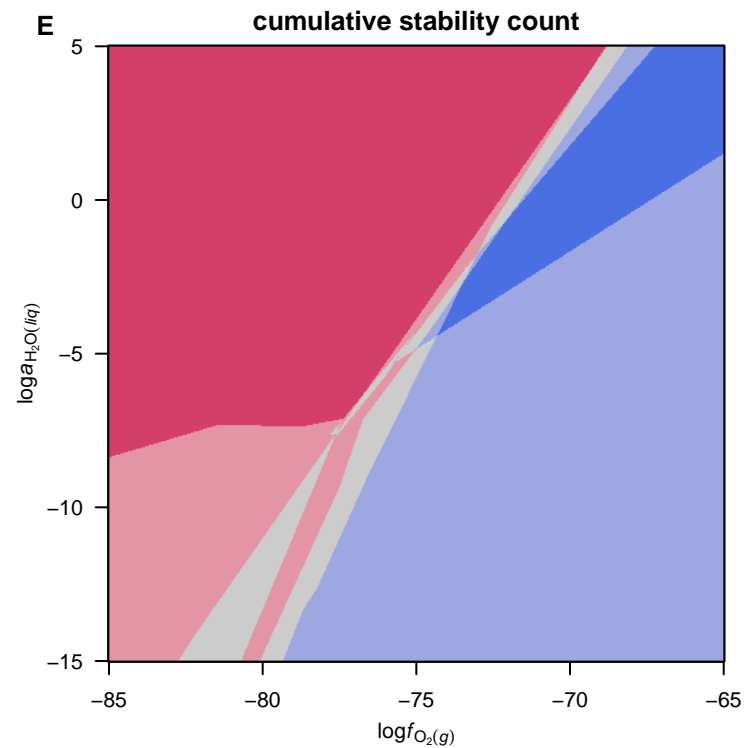

Supplement: Figure S3 [file peerj-04-2238-s004.pdf]
